# Revised Genome Sequence of *Staphylococcus aureus* Bacteriophage K

**DOI:** 10.1128/genomeA.01173-13

**Published:** 2014-01-23

**Authors:** Jason J. Gill

**Affiliations:** Department of Animal Science and Center for Phage Technology, Texas A&M University, College Station, Texas, USA

## Abstract

Bacteriophage K is a member of the virulent Twort-like group of myophages infecting *Staphylococcus aureus*. The revised sequence presented here includes 12,436 bp of additional sequence not present in the previously available phage K genome (GenBank accession no. NC_005880) and updated annotations, and has been reopened at the predicted terminal repeat boundary.

## GENOME ANNOUNCEMENT

*Staphylococcus aureus* is a Gram-positive opportunistic pathogen, many strains of which are antibiotic resistant (1). Among the bacteriophages capable of infecting *S. aureus*, members of the Twort-like group possess broad host ranges and a virulent lifestyle that makes them attractive as potential therapeutics against this pathogen. Phage K is among the best-studied members of this group, the genome of which was initially published in 2004 (accession no. NC_005880) (2).

Bacteriophage K (ATCC 19685-B1) was obtained from the ATCC and routinely propagated on *S. aureus* strain Newbould 305. Phage genomic DNA was extracted using previously described methods (3) and sequenced in an Illumina MiSeq 250-bp paired-end run with a 500-bp insert library at the University of Texas Genomic Sequencing and Analysis Facility, Austin, TX. Quality-controlled and trimmed sequences were assembled with Velvet version 1.2.10 (4) to produce a final assembly of 139,831 bp at 30-fold average coverage. Gene prediction was conducted with Glimmer (5) and GeneMark.hmm (6), followed by manual curation. Functional annotations were assigned using a combination of BLASTp (7) and InterProScan version 4.7 (8). Where applicable, the annotation was based on the published phage K annotation, including previously described introns (2).

The phage K unit genome presented here is 139,831 bp in length and contains two large regions of additional sequence not present in the published phage K genome (GenBank accession no. NC_005880) (2). The first, a 2,888-bp region, comprises bases 4191 to 7078, containing genes 11 to 16. A second region of 9,546 bp comprises bases 123190 to 132735 and contains genes 169 to 196. A third 2-bp insertion comprises bases 3087 to 3088 in a noncoding region. The 9,546-bp insertion occurs within K_ORF108 (YP_024536), altering the C terminus of this gene. Other than these differences, the remainder of the phage K genome sequence reported here was found to be identical to the previously reported phage K DNA sequence. The 12,436 bp of new sequence was predicted to contain 35 protein-coding genes. An additional 62 previously unannotated genes were added to the record, bringing the total number of predicted protein-coding genes in phage K to 212 and the coding density to 90.2%. Four tRNA genes were also annotated, encoding predicted Met, Trp, Phe, and Asp tRNAs.

Based on a recent analysis of Twort-like phages, phage K is predicted to possess a terminally redundant nonpermuted genome as demonstrated for other members of the SPO1-like family (9). The phage K genome was reopened at the predicted terminal repeat (TR) boundary based on sequence similarity to the TR boundaries of other Twort-like phages (9) and experimental analysis of a related *S. aureus* bacteriophage (unpublished data). Phage K is predicted to contain an 8,486-bp terminal redundancy, making the entire packaged genome 148,317 bp in length.

### Nucleotide sequence accession number.

This genome sequence was deposited into GenBank under accession no. KF766114.
